# From ‘Omics to Otoliths: Responses of an Estuarine Fish to Endocrine Disrupting Compounds across Biological Scales

**DOI:** 10.1371/journal.pone.0074251

**Published:** 2013-09-25

**Authors:** Susanne M. Brander, Richard E. Connon, Guochun He, James A. Hobbs, Kelly L. Smalling, Swee J. Teh, J. Wilson White, Inge Werner, Michael S. Denison, Gary N. Cherr

**Affiliations:** 1 Bodega Marine Laboratory, University of California Davis, Bodega Bay, California, United States of America; 2 Department of Environmental Toxicology, University of California Davis, Davis, California, United States of America; 3 Department of Biology and Marine Biology, University of North Carolina Wilmington, Wilmington, North Carolina, United States of America; 4 Department of Anatomy, Physiology, and Cell Biology, School of Veterinary Medicine, University of California, Davis, Davis, California, United States of America; 5 Department of Wildlife, Fisheries, and Conservation Biology, University of California Davis, Davis, California, United States of America; 6 United States Geological Survey, Sacramento, California, United States of America; 7 Swiss Centre for Applied Ecotoxicology, Eawag/École Polytechnique Fédérale de Lausanne, Duebendorf, Switzerland; 8 Departments of Nutrition, University of California Davis, Davis, California, United States of America; Glasgow Caledonian University, United Kingdom

## Abstract

Endocrine disrupting chemicals (EDCs) cause physiological abnormalities and population decline in fishes. However, few studies have linked environmental EDC exposures with responses at multiple tiers of the biological hierarchy, including population-level effects. To this end, we undertook a four-tiered investigation in the impacted San Francisco Bay estuary with the Mississippi silverside (*Menidia audens*), a small pelagic fish. This approach demonstrated links between different EDC sources and fish responses at different levels of biological organization. First we determined that water from a study site primarily impacted by ranch run-off had only estrogenic activity *in vitro*, while water sampled from a site receiving a combination of urban, limited ranch run-off, and treated wastewater effluent had both estrogenic and androgenic activity. Secondly, at the molecular level we found that fish had higher mRNA levels for estrogen-responsive genes at the site where only estrogenic activity was detected but relatively lower expression levels where both estrogenic and androgenic EDCs were detected. Thirdly, at the organism level, males at the site exposed to both estrogens and androgens had significantly lower mean gonadal somatic indices, significantly higher incidence of severe testicular necrosis and altered somatic growth relative to the site where only estrogens were detected. Finally, at the population level, the sex ratio was significantly skewed towards males at the site with measured androgenic and estrogenic activity. Our results suggest that mixtures of androgenic and estrogenic EDCs have antagonistic and potentially additive effects depending on the biological scale being assessed, and that mixtures containing androgens and estrogens may produce unexpected effects. In summary, evaluating EDC response at multiple tiers is necessary to determine the source of disruption (lowest scale, i.e. cell line) and what the ecological impact will be (largest scale, i.e. sex ratio).

## Introduction

Endocrine disrupting chemicals (EDCs) agonize, antagonize or synergize the effects of endogenous hormones and are known to cause a number of physiological and behavioral abnormalities in fishes [1]. EDCs originate from a variety of sources, such as treated wastewater effluent and agricultural, ranch, or urban run-off [2], [3], and are widespread in the aquatic environment [1], [4]. Examples of hormonal disruptions in fishes produced by EDCs include altered secondary sexual characteristics, males producing egg proteins (vitellogenin, choriogenin), and reduced sperm quality [5], [6], [7]. Both theoretical and empirical data indicate that EDCs can also cause declines in fish populations [8], [9].

Recent studies have utilized the results from single-EDC laboratory exposures to produce predictive population models [10], [11], to assess multiple genomic and organismal level endpoints in response to known environmental mixtures [12], and to link EDC-perturbations in gonad or gene expression changes in fish with reduced reproductive performance or varying degrees of urbanization or agricultural activity [2], [13], [14]. However, to date no single study has attempted to link exposure to different environmental EDC mixtures, such as urban and ranch run-off, with responses at multiple tiers of the biological hierarchy, including population-level effects, within one study system. Linking molecular level responses (i.e. mRNA levels) with higher level effects (i.e. sex ratio) at sites exposed to different sources of EDCs may help to better determine the predictive value of biomarkers (i.e. vitellogenin).

A second limitation of many environmental EDC investigations is the use of model fish species that are not necessarily ecologically relevant. Most EDC studies continue to use several common laboratory denizens to assess impacts (e.g., zebrafish – *Danio rerio*, medaka *– Oryzias latipes*, fathead minnow – *Pimphales promelas*) [15], [16], [17]. As a result, assumptions about sensitivity to EDCs are primarily based on these few species and relying on a limited number of fish species to represent responses across a range of taxa may lead to an underestimation of toxicity [18]. This is of particular concern when considering threatened or endangered species. In this situation the use of a resident fish as a surrogate may be a better alternative to evaluating the response instead of typically utilized lab species.

The San Francisco Bay (SFB) estuary, the largest Pacific estuary in North or South America, is ecologically critical [19], subject to a diverse array of anthropogenic inputs including EDCs [20], [21], [22], and is home to a number of declining fish species [23]. It is an example of an ecosystem in need of a surrogate species to evaluate potential EDC impacts. To date, however, a study on both estrogenic and androgenic endocrine disruption has yet to be conducted on fishes in the SFB estuary. Recently, awareness of EDC prevalence has increased, with estrogenic activity documented in the watershed’s rivers [21] and agricultural drain water [22]. Discerning impacts on SFB fishes, however, is challenging because the region’s many highly impacted native species cannot be collected in large enough numbers due to population decline [23]. Selection of *Menidia audens* (Mississippi silverside, Atherinidae), introduced in the early 1970s, as a surrogate for EDC studies is highly appropriate as it is distributed through the entire estuary and shares life history traits such as habitat use, diet and short lifespan (1–2 years) with some endangered fishes [24]. Furthermore, it has been confirmed that *Menidia* species closely related to *M. audens* have a combination of genetic and temperature sensitive sex determination, in many cases following a seasonal pattern of spring female-biased sex ratios and summer to fall male-biased ratios [25], [26]. The adaptive significance of this pattern is that females have more time to grow larger and hence have the ability to carry more eggs [27], [28]. Few EDC studies have been performed with fish that have temperature sensitive sex determination (TSD), but recent findings indicate that *Menidia* species are highly sensitive to EDCs [29], [30], that exposure may disrupt the adaptive benefits of TSD [31], and that the potential for *Menidia* species to be widely-utilized North American estuarine bioindicators is unparalleled [29], [32].

The use of markers from several different levels of biological organization using a resident model fish allows for inferences to be made about the overall impact on the reproductive health and potential population consequences for that species. To this end, we undertook a four-tiered investigation into estrogenic and androgenic EDC effects on *M. audens*. Our main objective was to integrate observations at each biological scale in order to determine whether the reproductive health of *M. audens* was being negatively impacted by sites receiving different non-point sources of EDCs (urban vs. ranch run-off), and if so what the mechanism(s) of endocrine disruption may be. Our investigation spanned four tiers of increasing levels of biological organization: 1) We measured overall estrogenic and androgenic activity in the water column at each site using recombinant cell lines containing an estrogen- or androgen-sensitive reporter gene and determined via chemical analysis whether particular hormones, alkylphenols, and pesticides were present. 2) At the molecular level, we quantified changes in mRNA levels of endocrine-related genes in fish. 3) At the whole-organism level, we examined differences in gonadal somatic index, length, and growth rate. 4) At the population level, we measured the sex ratio over of the course of two spawning seasons. Ultimately our research approach could be applied to EDC investigations in other North American estuaries, the majority of which contain *Menidia* species [32].

## Methods

### Site Selection

**Figure 1:**
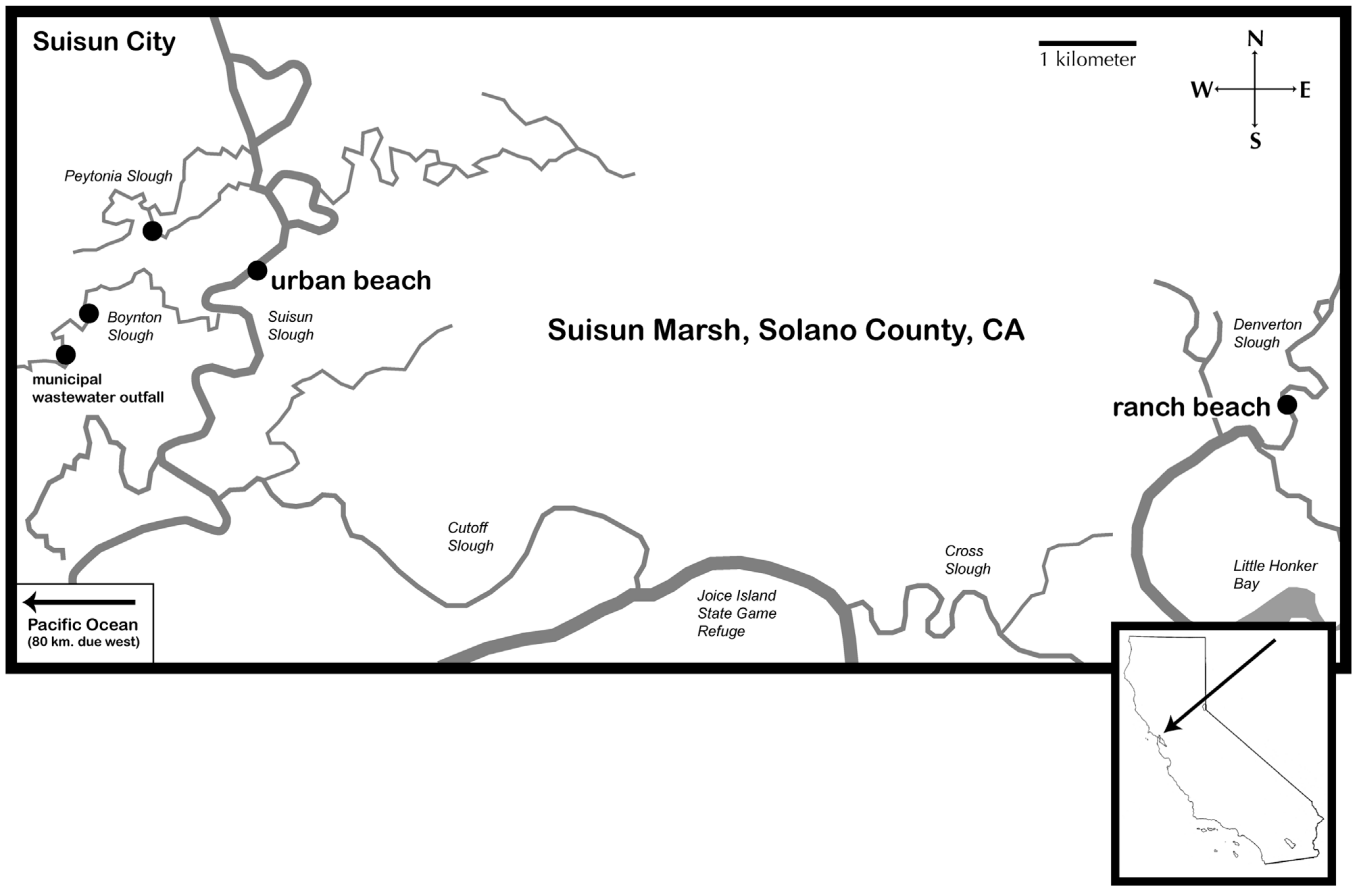
Map of study sites, Suisun Marsh, San Francisco Bay. Suisun Marsh is located approximately 96 kilometers NE of San Francisco (SF) Bay. Water samples were collected for reporter gene assay and hormone measurement from Boynton Slough (at outfall and 300 m downstream), Peytonia Slough, Suisun Slough (the urban beach) and Denverton Slough (the ranch beach). Passive sampling polyethylene devices (PEDs) were deployed at the outfall and the ranch beach. Fish were collected via beach seine from the urban and ranch beaches.

Two seining beaches were selected based on knowledge of *M. audens* occurrence and differences in the major class of EDCs present. Ultimately we sought to compare the response to urban EDCs with that of EDCs typically found in ranch run-off in *M. audens* populations living in similar environmental conditions (defined by tidal regime, salinity, temperature). We seined *M. audens* monthly from a primarily urban-influenced beach (38° 13′ 5.47″ N, 122° 1′ 48.50″ W) in Suisun Slough, Suisun Marsh (hereafter referred to as “urban”) and a primarily ranch-influenced beach (38° 11′ 56.76″ N, 121° 54′ 39.31″ W) (Figure 1) in Denverton Slough, Suisun Marsh (hereafter referred to as “ranch”) in Solano County, California, USA. The “urban” beach receives run-off from Suisun City, CA (population = 28,330), treated effluent from the Fairfield-Suisun Sewer District outfall (tertiary treatment, 61 million l/day) located in Boynton Slough, and limited ranch run-off from the adjacent Rush Ranch (cattle), which is fenced off from the marsh. The “ranch” beach primarily receives run-off from a private cattle ranch that abuts and shares a beach with Denverton Slough. Although some exchange between these two sites may occur over long time periods, the metapopulations of silversides at each beach are relatively isolated from one another, separated by a distance of approximately 12 km. *Menidia audens* collection from both sites was performed with specific permission from the California Department of Fish and Game under Scientific Collecting Permit #10086.

### Fish Collection and Processing

Fish were collected monthly from the urban and ranch beaches from March through October of 2009 and 2010, as previously described [33]. All research was done in accordance with the University of California, Davis Institutional Animal Care and Use Committee (IACUC), under approved protocol #13353. Captured fish were kept in a cooler with aeration and transported back to the UC Davis Bodega Marine Lab, Bodega Bay, CA, for processing. During the 2009 sampling season approximately 20 fish from each site were kept alive and held in aquaria at 5–10 ppt salinity for 4–5 months to serve as depurated controls for gene expression analyses. The remaining fish were anesthetized in accordance with IACUC protocol #13353, sacrificed, and livers were immediately removed and snap-frozen on liquid nitrogen for RNA extraction. Gonads were removed, weighed, and fixed for 24 hours in Davidson’s solution [34] followed by storage in phosphate buffered 10% formalin. Fish length and sex were recorded prior to and following dissection, respectively. Fish mass was measured after gonad removal and used in addition to gonad mass to obtain a total mass for gonadosomatic index (GSI) calculation (GSI = gonad mass/total mass). Sagittal otoliths were extracted, mounted on slides, photographed, and growth increments were counted and measured based on previously described methods [35].

### Length, Sex Ratio, GSI

Because fish length, sex ratio, and GSI were expected to vary over the sampling period, we tested for differences among sites in those variables while including year and Julian date as covariates in a linear model (length) or logistic regression (sex ratio and GSI). Because no females were seined from the urban beach after July in either 2009 or 2010, GSI analysis was ended at that time point.

### Otolith Growth Rate Analysis

*Menidia* species lay down daily rings on their otoliths, which are calciferous structures in the inner ear that are used as gravity, balance, movement, and directional indicators. These structures have been used for decades to measure the growth rates of *Menidia* species and other fishes [36]. The width of daily otolith increments is proportional to daily somatic growth in *M. menidia* [36]. We examined daily otolith increments from ranch and urban fish sampled from March-September 2009. Only growth during the first growing season was examined because growth slows in winter, changing the relationship between otolith size and somatic size [35]. The onset of winter growth is indicated by a dark band; we only examined growth rings preceding that band.

Plots of otolith radius at each increment versus age indicated that all fish had approximately linear growth trajectories, so we used linear regression to model radius as a function of age. There was no evidence for seasonal effects on growth, so we pooled fish across collection dates to test for the effects of site and sex on growth rate. We fit linear regression models with a random effect for fish (thus controlling for the non-independence of increment widths within each fish [37]. We fit models with fixed effects for age, site, sex, and all of their interactions, then removed non-significant interaction terms (*p*>0.1) in a stepwise manner, as is standard practice [38]. Mixed-effects models were fit using function lme in the nlme package version 3.1 [39] for R; note this approach is equivalent to repeated-measures ANOVA for longitudinal data [37].

### Histology

Gonad tissue samples fixed in 10% (w/v) PBS buffered formalin were dehydrated in a graded ethanol series and embedded in paraffin. Tissue blocks were sectioned (4 µm thick) and stained with hematoxylin and eosin [34]. Tissue sections were examined under a BH-2 Olympus microscope for common and/or significant lesions. Lesions in testes were qualitatively scored on a scale of 0 =  not present, 1 =  mild, 2 =  moderate, and 3 =  severe [40]. Although ovaries were also sectioned, most were not of a high enough quality to be scored.

Ordinal necrosis ratings were converted into a binomial metric for analysis using logistic regression. The necrosis rating for each sample was classified as ≥1, ≥2, or ≥3; three separate logistic regressions were then used to determine whether the urban and ranch beaches differed in the proportion of observations in each of those categories. Because the data exhibited quasi-separation, models were fit using Firth’s bias-reduced logistic regression in the logistf package; R 2.11) [41].

### Total RNA Extraction and cDNA Synthesis

Total RNA was extracted using TRIzol Reagent (Invitrogen Corp., Carlsbad, CA) following the manufacturer’s protocols, followed by DNase digestion to remove any traces of genomic DNA. Total RNA concentrations were determined using a NanoDrop ND1000 Spectrophotometer (NanoDrop Technologies, Inc., Wilmington, DE, USA) and integrity was verified through electrophoresis on a 1% (w/v) agarose gel [42].

Complementary DNA (cDNA) was synthesized using 1 µg total RNA, with 50 units of Superscript III (Superscript III Reverse Transcriptase – Invitrogen, Carlsbad, CA, USA), 600 ng random primers, 10 units of RNaseOut, and 1 mM dNTPs (Invitrogen) to a final volume of 20 µl. Reactions were incubated for 50 min at 50°C followed by a 5 min denaturation step at 95°C. Samples were diluted 3-fold with the addition of 40 µl nuclease-free water to a total volume of 60 µl for subsequent real-time PCR assessments.

### Gene Expression

Primer pairs and fluorescent probes for real-time TaqMan® PCR (measurement of mRNA levels) were designed using Roche Applied Science Universal Probe Library Assay Design (Table 1). Real-time TaqMan PCR was conducted as described in Connon et al [43] using an automated fluorometer (ABI HT 7900 A FAST Sequence Detection System, Applied Biosystems). SDS 2.2.1 software (Applied Biosystems) was used to quantify transcription. GAPDH was identified using GeNorm [42] as a suitable reference gene for this assessment. Quantitative PCR data was analyzed using the relative quantification log_2_^(-delta delta Ct)^ method [44]. Data are reported as the log_2_ gene transcription relative to GAPDH and normalized to the mean transcription of each gene corresponding to the experimental depurated controls from each site to allow for direct comparison between the assessed sites.

**Table 1:** Primer and probe sequences of genes used as molecular biomarkers to assess the impact of Endocrine Disrupting Chemicals on *M. audens*.

Transcript Name	Primer Sequence	Roche Probe Number
Fwd: *M.audens* estrogen receptor 1	ACGCTTCCGCATGCTCA	#15
Rev: *M. audens* estrogen receptor 1	CTCCATTGTGCCAGTGCAGA	
Fwd: *M. audens* estrogen receptor 3	CATTATGCCCTCCACGCACT	#52
Rev: *M. audens* estrogen receptor 3	GACCATCCTGGGAAACTGATCTT	
Fwd: *M. audens* androgen receptor x	ATCCGCATGCAGTGCTCATA	#31
Rev: *M. audens* androgen receptor x	CCCCAGACCTCGTATTCAACG	
Fwd: *M. audens* choriogenin L	CATCCAGTCATCAGTCATGAGTTTC	#82
Rev: *M. audens* choriogenin L	GGTCCCGTTTTCTGCAGTTAAG	
Fwd: *M. audens* thyroid receptor alpha	TGTCGGACGCCATATTCGAT	#51
Rev: *M. audens* thyroid receptor alpha	CCTCGGTGTCATCCAAGTTGA	
Fwd: *M. audens* GAPDH	GGTGGTGAACACACCAGTGG	#159
Rev: *M. audens* GAPDH	CACGAGAGGGACCCAACTAACA	
Fwd: *M. audens* Vtg	GTAGAGTTCATGAAGCCCATGCT	#108
Rev: *M. audens* Vtg	AAATCAATGTAAGCGGCAAAGG	
Fwd: *M. audens* insulin-like growth factor 2	GGCTGCCTTCCTATTCCACAC	#38
Rev: *M. audens* insulin-like growth factor 2	GCAGGTCATACCCGTGATGC	

Primer pairs and fluorescent probes for real-time TaqMan PCR were designed using Roche Applied Science Universal Probe Library Assay Design.

Differences in gene expression (i.e. mRNA levels) between sites were assessed using *t*-tests on normalized data. We report results as fold-change in expression, which we calculated from normalized data using the log_2_^(-delta *Ct*)^ method [45]. Genesis software version 1.7.5 software [46] was used to generate an agglomerative hierarchical clustering heat map representing relative changes in transcript levels. This program uses a hierarchical algorithm to aggregate similarly expressed genes and expression patterns.

### Water Sample Collection

Water samples for chemical and CALUX analyses were collected at the outfall in Boynton Slough and approximately 300 meters downstream of the outfall, in an adjacent slough not directly receiving treated effluent, and from the urban beach and the ranch beach. Samples were collected from 30–60 cm below surface in I-Chem 200 series 1 l amber glass bottles from 5 sites: urban slough (38° 13′ 16.08″ N, 122° 2′ 52.86″ W), urban beach (38° 13′ 5.47″ N, 122° 1′ 48.50″ W), ranch beach (38° 11′ 56.76″ N, 121° 54′ 39.31″ W), wastewater treatment outfall (38° 12′ 30.60″ N, 122° 3′ 25.26″ W), and downstream of outfall (38° 12′ 30.66″ N, 122° 3′ 12.54″ W). Bottles were rinsed with sample water once before filling, leaving as little head-space as possible. Samples were then kept on ice in coolers until extraction for either chemical or cell line analyses <24 h later.

### CALUX

The CALUX mammalian cell bioassay utilizes a human ovarian carcinoma (BG-1) cell or breast cancer (TD47-D) cell line, which has been stably transfected with an estrogen-responsive or androgen-responsive luciferase reporter plasmid, respectively. These CALUX cell lines respond to estrogenic (BG-1) or androgenic (TD47-D) chemicals with the induction of expression of firefly luciferase proportional to activation of the estrogen or androgen receptor (ER or AR) signaling pathways [47], [48]. Preparation of extractions from water grab samples to be incubated with the BG-1 and TD-47 cells were performed according to methods previously described [49]. Samples collected in fall 2009 were concentrated 2500× and exchanged into dimethylsulfoxide (DMSO), while samples collected in spring 2010 were concentrated 4500×. Spring samples were more highly concentrated due to expected dilution from Northern California’s typically higher winter rainfall relative to spring-summer. Samples suspended in DMSO were capped and stored frozen at −20°C until being evaluated for estrogenic, androgenic, anti-estrogenic and anti-androgenic activity using recombinant cell bioassays as previously described [48]; additional details given in Appendix S1.

CALUX concentration-response curves obtained using the positive control hormones 17β-estradiol (concentrations of 1×10^–15^ to 1×10^–6^ M) or testosterone (concentrations of 1×10^–12^ to ×10^–5^ M) were fit using logistic regression with binomial error and logit link [50]. These standard curves were then used to estimate the relative equivalent concentration of estrogenic or androgenic chemicals in each environmental sample analyzed with CALUX. Confidence intervals (95%) on the equivalent concentrations were estimated using a Monte Carlo approach [51]: we used the means and covariances of the logistic model coefficients to simulate a distribution of 1000 different values of those coefficients; we then used that distribution to simulate a distribution of the equivalent hormone concentration for each CALUX sample. Differences among sites were tested using ANOVA followed by Tukey test and data were log-transformed in order to ensure homogeneity of variances.

### Chemical Analysis

#### Steroids and alklyphenols in surface water

Methods for the extraction of water grab samples for steroid and alkylphenol measurement were performed as previously described [52].

#### Pesticides in surface water

Surface water samples (1 l) were filtered using 0.7 µm glass fiber filters (GF/F) (Whatman, Florham Park, New Jersey), extracted onto Oasis HLB solid-phase extraction (SPE) cartridges (6 ml (volume), 500 mg (substrate amount), 60 µm (sorbent particle size), Waters Corporation, Milford, Massachusetts), dried, eluted with ethyl acetate, reduced to 200 µl and analyzed for a suite of 56 pesticides by gas chromatography –mass spectrometry operating in electron ionization mode (GC-EIMS). Prior to extraction, samples were spiked with ^13^C_3_-atrazine, and diazinon diethyl-d_10_ (Cambridge Isotopes, Andover Massachusetts) as recovery surrogates [53].

#### Pesticides in polyethylene devices

Low density polyethylene devices (PEDs) were deployed approximately 60 cm below the water’s surface at the municipal wastewater outfall and the ranch beach in polypropylene holders for a period of 14–19 d, then removed and placed on ice until extraction. The PED membranes (Brentwood Plastics, Brentwood, MO; 70±1 µm (pore size)) were pre-cleaned by soaking in dichlormethane (DCM) for 48 h followed by methanol (MeOH) for 24 h and finally deionized water for 24 h. PEDs were stored in glass jars in deionized water prior to use to minimize the effects of airborne laboratory contaminants. Field-deployed PEDs were extracted based on methods modified from published sources [54]. Prior to extraction, PEDs were rinsed with deionized water and wiped with a damp Kim-wipe to remove any debris and biofouling. PEDs were spiked with 100 µl of a 2 ng/µl solution of ring-^13^C_12_-*p,p’* DDE and phenoxy-^13^C_6_-*cis*-permethrin used as recovery surrogates and extracted twice with 60 ml of DCM using a sonicator bath for 30 minutes each. The sample extracts were combined, dried over sodium sulfate (Na_2_SO_4_), reduced to 0.5 ml using a Turbovap II evaporation system (Biotage LLC, Charlotte, NC) and analyzed for 56 pesticides using GC-EIMS. Details on the determination of detection limits, the instrumental analysis performed, and quality assurance standards are provided in Appendix S1.

### Data Analysis

Unless otherwise indicated, all calculations were performed using R version 2.11 [55]. Results were considered to be significant at a *p*≤0.05, unless otherwise indicated.

## Results

### Sex Ratio

**Figure 2:**
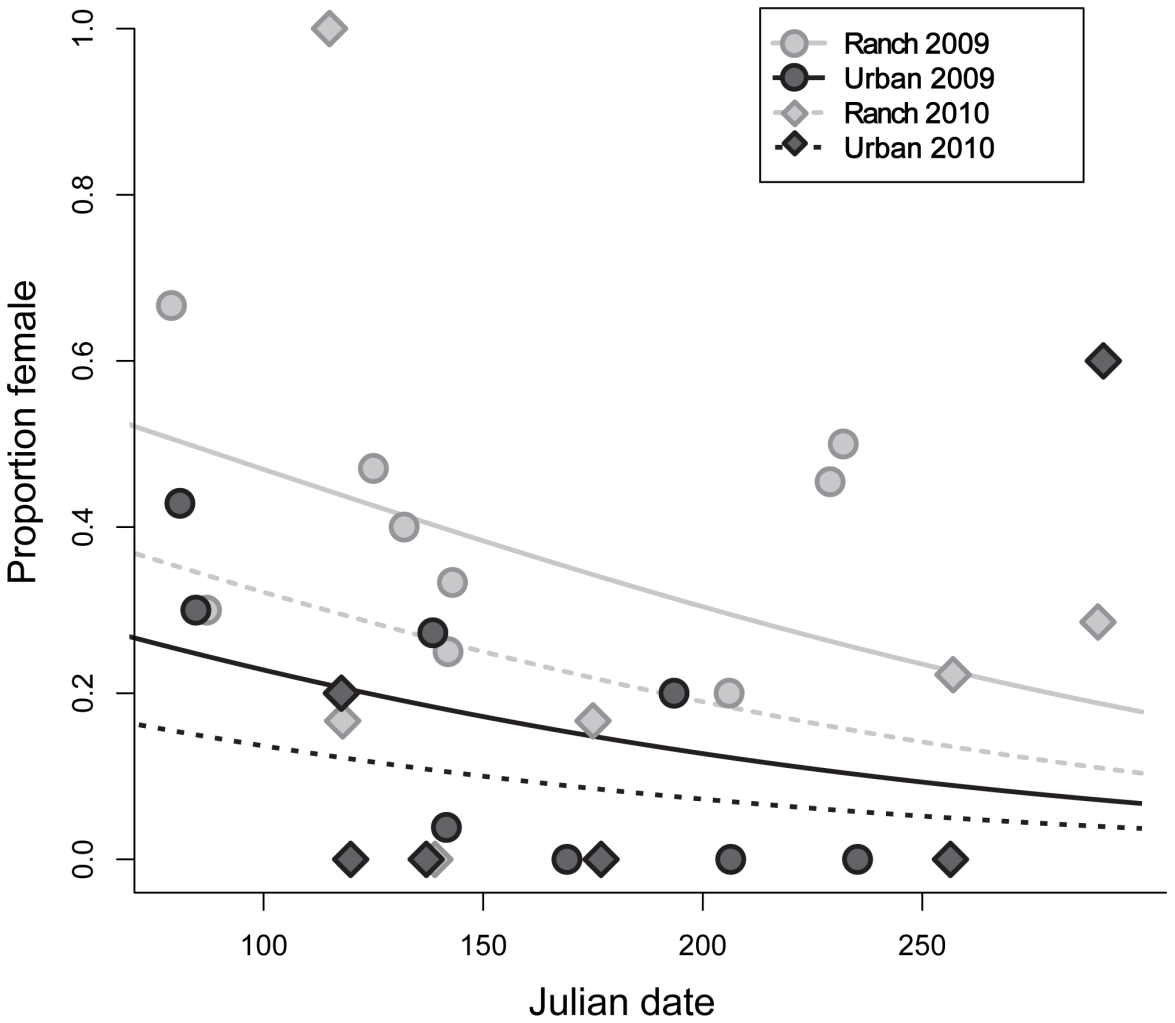
Sex ratio by site and year. Points indicate sex ratio (proportion female) of silversides collected at the ranch and urban beach in 2009 and 2010 on the indicated sampling dates. Curves are logistic regression fits.

The proportion of female fish caught from the urban beach was significantly lower than the proportion caught at the ranch beach in both 2009 and 2010, and a lower proportion of female fish was caught at both beaches in 2010 compared to 2009 (Table S1, Figure 2). While the observed sex ratio in early spring ranged from 38–52% at the ranch beach in 2009 and 2010, respectively, the observed sex ratio at the urban beach ranged from 18–27%.

### Gonadosomatic Index

**Figure 3:**
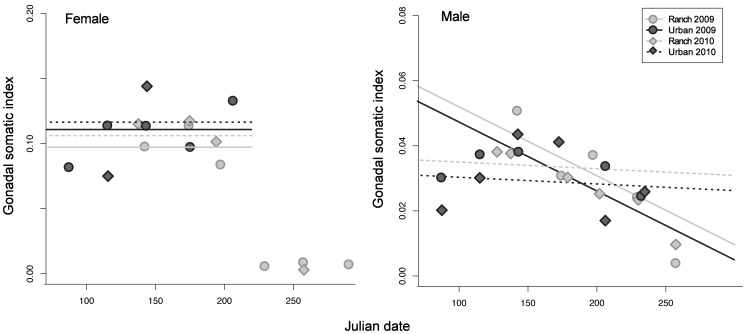
Variation in gonadosomatic index (GSI) by sex, site, and year. Points indicate the GSI of female (left panel) and male (right panel) fish collected at the ranch and urban beachs on the indicated sampling dates. Curves are linear regression fits; there was no significant effect of Julian date in female fish, and data collected after day 220 were excluded from the regression because no females were collected at the urban site.

The gonadosomatic index (GSI) was significantly higher in males caught at the ranch beach than in males caught at the urban beach in both 2009 and 2010 (Table S2, Figure 3). Male GSI was significantly lower in 2010 than in 2009, and decreased over time from March to October; this decrease was significantly faster in 2009 than 2010 (Table S2, Figure 3). No significant difference was observed between the GSI of urban and ranch females (Table S3), and there was no change in female GSI over time (Table S3, Figure 3).

### Growth

**Figure 4:**
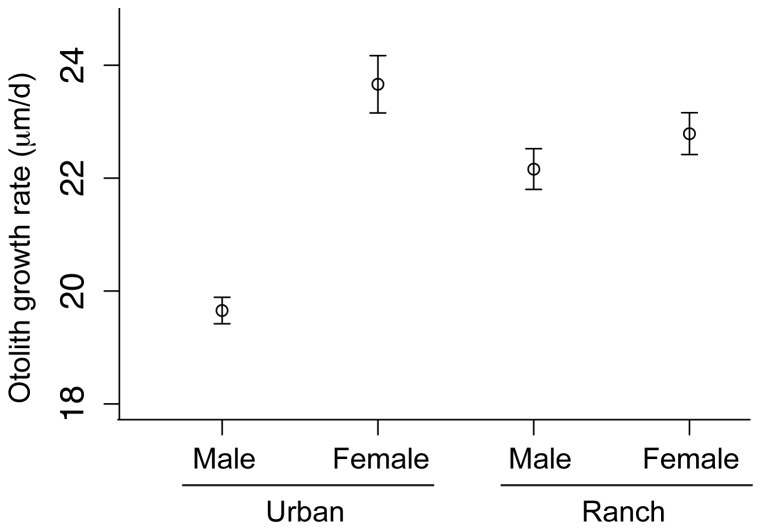
Variation in otolith growth rates by site and sex. Average growth rates for male and female silversides were estimated at each site using otolith increment analysis (*n* = 47 urban males, 10 urban females, 26 ranch males, 19 ranch females). Error bars represent 95% confidence intervals.

Otolith radius increased with fish age (as expected) but there were significant effects of both site and sex on that growth rate. Urban males grew slower than females (significant negative sex × age interaction; Table S4), and fish from the urban site grew overall slightly faster than those from the ranch site (significant positive site × age interaction; linear regression; *p*<0.05; Table S4), but males from the urban site grew much more slowly than all other fish (significant negative site × sex × age interaction; Table S4). To visualize differences in otolith growth rate, we combined significant model terms to obtain the predicted change in otolith size by age for each site and sex (Figure 4). In the mixed-effect model, the random effect of individual fish accounted for 24% of total variance in otolith radius.

There were also significant sex × site effects on overall otolith size; otoliths from the ranch site were significantly larger than urban otoliths, but urban male otoliths were significantly larger than other otoliths (Table S4). These effects on overall otolith size did not affect otolith growth rates. There was no evidence of a decrease in growth rate with increasing age over the first growing season, so the differences in the final ages of sampled fish did not bias estimates of growth.

### Standard Length

**Figure 5:**
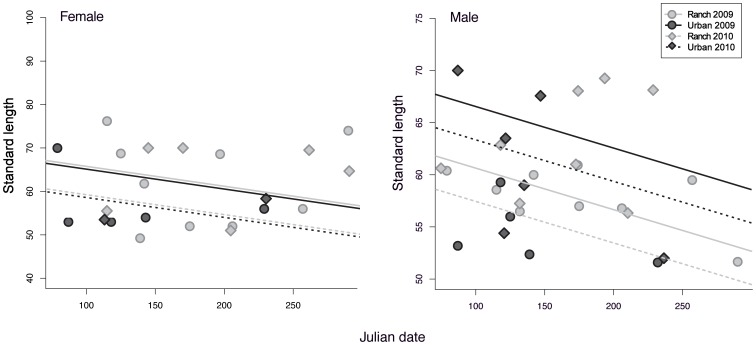
Variation in fish length among sites, sexes and years. Points indicate the standard length (SL) of female (left panel) and male (right panel) fish collected at the ranch and urban beachs on the indicated sampling dates. Curves are linear regression fits.

Male fish caught at the urban beach were significantly longer than males at the ranch beach (Table S5). Length of male fish also decreased over time, and males caught in 2010 were significantly smaller than those caught in 2009 (Table S5, Figure 5). By contrast, there was no significant difference in length between females captured at the two sites, and although there were trends towards decreasing size over time and smaller size in 2010 relative to 2009 (as in the male fish), those relationships were not significant (Table S6; Figure 5).

When sexes were analyzed together, females were significantly larger than males at the ranch beach, but unexpectedly, females were significantly smaller than males from the urban beach in both years (significant negative sex × site interaction; Table S7). There was also a decrease in size over time and smaller overall size in 2010 in this combined analysis (Table S7).

### Histology

**Figure 6:**
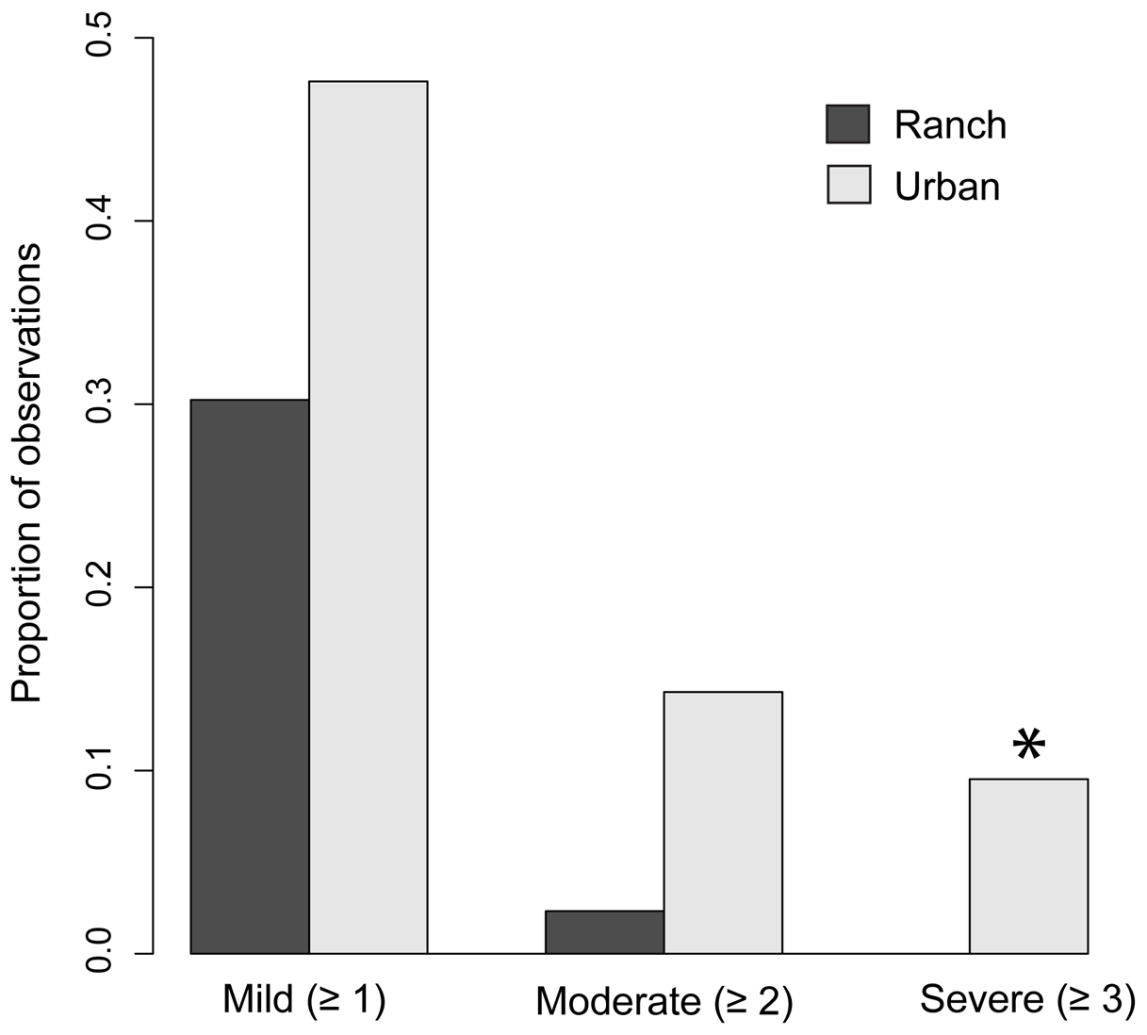
Histological evaluation of testes by site. Testes sectioned from male silversides collected at the ranch site (*n = *43) and urban site (*n = *42) in 2009 and 2010 were evaluated for necrosis and qualitatively scored on a scale of 1 =  mild, 2 =  moderate, and 3 =  severe. Bars show the proportion of males exhibiting a necrosis rating as bad or worse than the indicated score (≥1, ≥2, or ≥3). Asterisk indicates a significant difference between sites (bias-reduced logistic regression; *p*<0.05).

**Figure 7:**
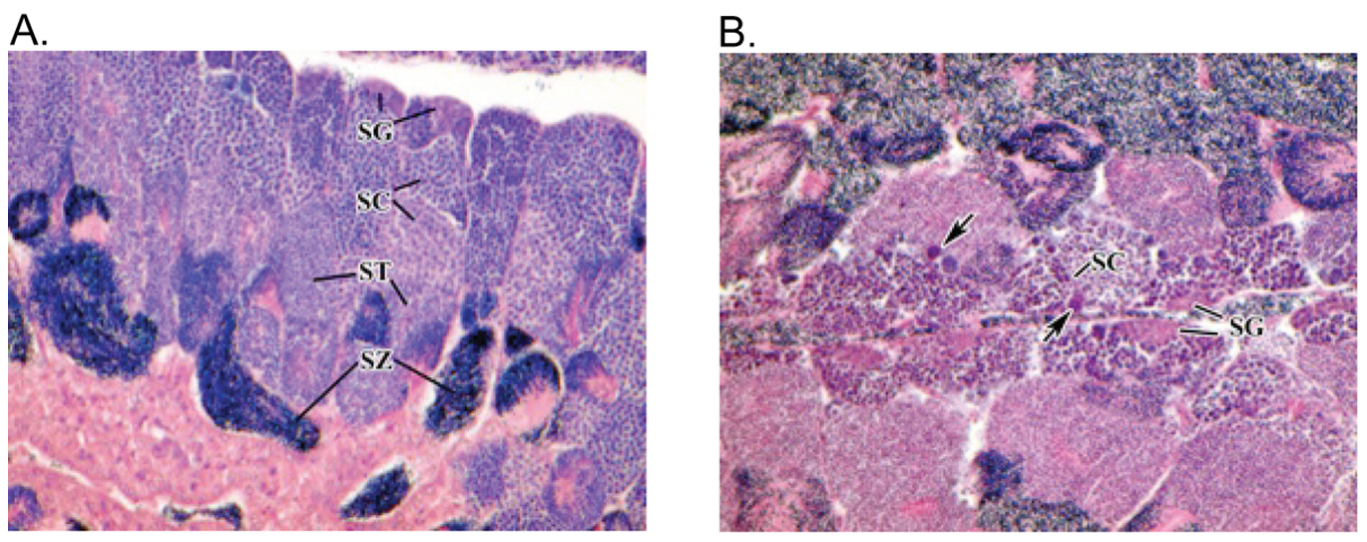
Comparative testicular morphology. A) Normal testicular morphology of male silverside collected at the ranch site; B) abnormal testicular morphology of male collected at the urban site showing severe germ cell necrosis (arrows) at the spermatocyte (SC) stage. Samples prepared in H&E paraffin section. SG = spermatogonia; SC = primary and secondary spermatocytes; ST =  spermaids; and SZ = spermatozoa.

The proportion of observations of severe necrosis (rating ≥3) was significantly higher in males caught at the urban beach than at the ranch beach (there were no rating ≥3 individuals observed at the latter site; Figure 6). There were not significant differences between the two sites in the proportions of observations of at least mild or at least moderate germ cell necrosis (rating ≥1 or ≥2); although in both cases the trend was towards higher necrosis at the urban site. No cases of intersex were observed. A representative micrograph of a normal testis and a severely necrotic testis are shown in Figure 7.

### Gene Expression

**Figure 8:**
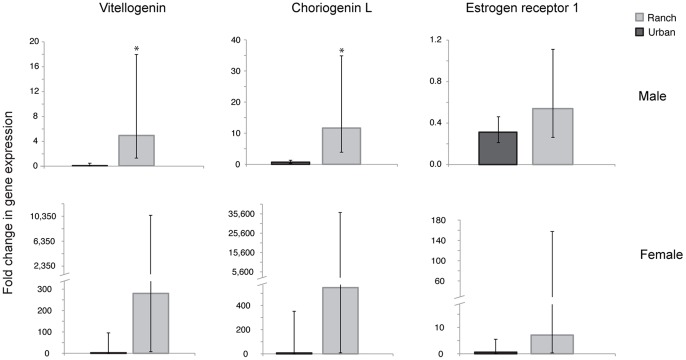
Differential expression of estrogen sensitive transcripts by site and sex. Expression of vitellogenin, choriogenin L, and estrogen receptor 1 (ESR1) was assessed using qPCR for male and female silversides collected at ranch (*n* = 11 males, 6 females) and urban (*n* = 11 males, 6 females) sites. Bars indicate mean fold change in expression relative to a reference gene (GapDH); error bars represent 95% confidence intervals. Asterisks indicate significant differences between sites (*p*<0.05).

**Figure 9:**
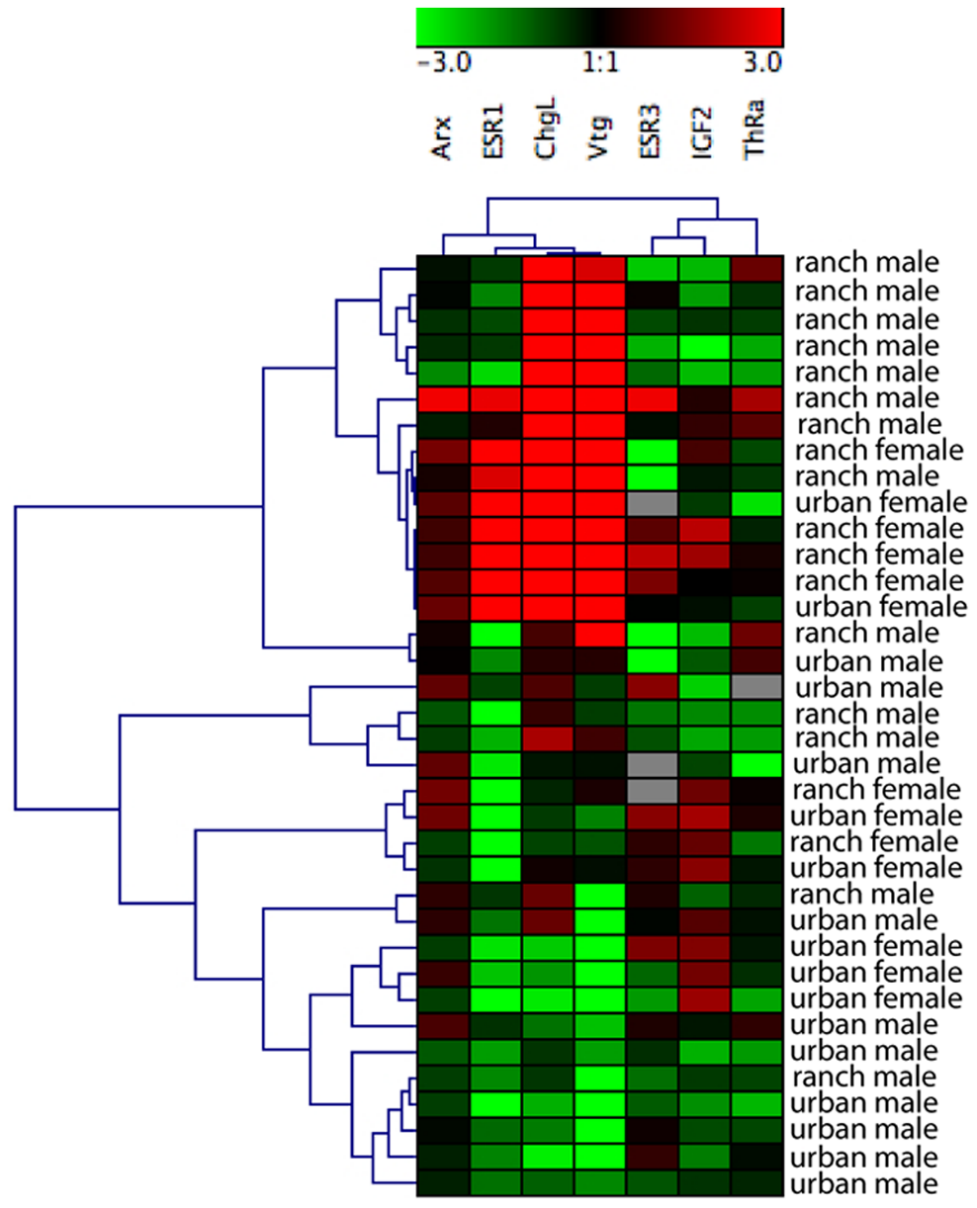
Heat map of transcript expression by site and sex. Expression of endocrine responsive genes (Arx, ESR1, ChgL, Vtg, ESR3, IGF3, and ThRa) was assessed using qPCR for male and female silversides collected at ranch and urban sites. Color indicates fold change in gene expression relative to a reference gene; red = upregulation, green = downregulation. Gray indicates missing data. Individual samples are ordered according to the results of a hierarchical cluster analysis.

Transcripts (mRNA levels) for *vitellogenin* (*Vtg*) and *choriogenin L* (*ChgL*), genes induced by estrogen, were significantly higher in males at the ranch site than in males at the urban site (*t*-test, *n* = 11, *p*<0.05; Figure 8). *Estrogen receptor 1* (*ESR1*) mRNA levels in males were not significantly different between the two sites (*t*-test, *n* = 11, *p*>0.05), although the trend was also towards higher expression in ranch males (Figure 8). Expression of the same three estrogen-related genes (mRNA levels) in females (*Vtg, ChgL*, and *ESR1*) was not significantly different between the two sites (*t*-test, *n* = 6, *p*>0.05). However, we attribute this lack of significance to the much greater variance around the mean in females; the mean fold difference in expression between the two sites was actually much greater than in males, and followed the same pattern of higher expression at the ranch site (Figure 9). No significant differences were found between the ranch and urban sites in either males or females in mRNA levels for the following genes: *thyroid receptor alpha* (*TRa*), *insulin-like growth factor 2* (*IGF-2*), *androgen receptor X* (*ARx*), or *estrogen receptor 3* (*ESR3*) (*t*-test, *n* = 6 (females), 11 (males), *p*>0.05). However, expression of all genes measured (*Vtg, ChgL, ESR1, ESR3, ARx, IGF-2, TRa*) generally clustered together by site overall (Figure 9). Ranch males and urban males clustered together closely, indicating similar levels of gene expression, while response in females at both sites was more variable.

### CALUX

**Figure 10:**
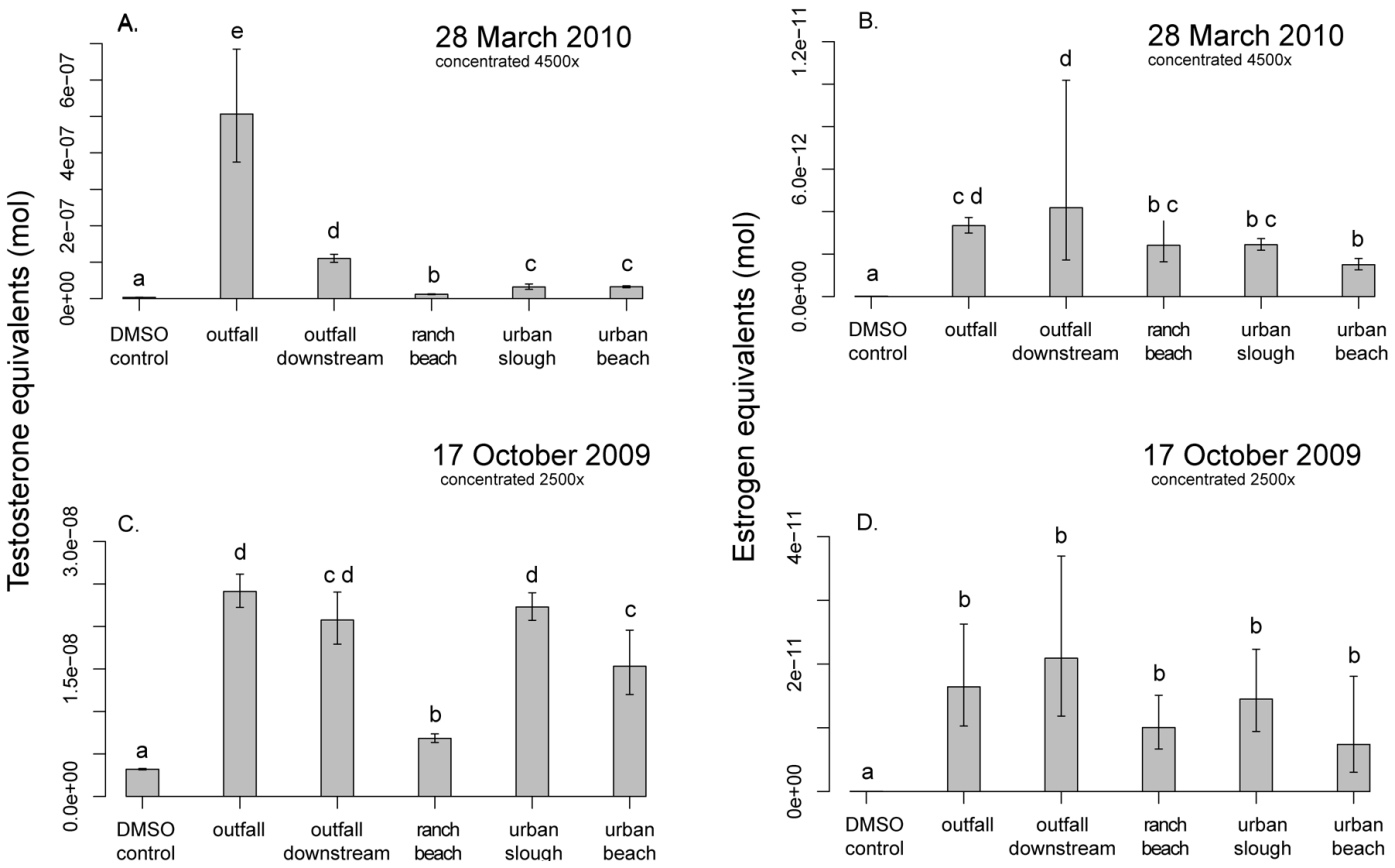
Estrogen and testosterone equivalents measured by CALUX. Estrogen and testosterone activity (measured as mole equivalents) were measured in water grab samples collected from the urban beach (Suisun Slough), urban slough (Peytonia Slough), ranch beach (Denverton Slough), wastewater outfall and downstream of outfall (Boynton Slough) on 17 Oct 2009 and 28 Mar 2010. Samples collected on 17 Oct 2009 were concentrated 2500× and those collected on 28 Mar 2010 were concentrated 4500×, and both resuspended in the control solvent DMSO. Bars indicate mean activity; error bars represent 95% confidence intervals. Treatments that are not significantly different (Tukey test; *p*>0.05) share the same letter.

Estrogenic activity was detected above solvent control (DMSO) levels in all water samples collected from Suisun Marsh (Figure 10B,D). Although samples from spring 2010 were more highly concentrated (4500×) than those collected in fall 2009 (2500×), estrogen equivalents were lower at all sites sampled in spring 2010. On both sampling dates, the sample taken 300 m downstream of the outfall was highest in estrogen equivalents, although not significantly different from the outfall in spring 2010 and not significantly different from any of the other sites on 17 October 2009.

Androgenic activity was also detected above solvent control (DMSO) levels at all sites sampled in fall 2009 and spring 2010 (Figure 10A,C). In contrast to measured estrogen equivalents, testosterone equivalents at all sites sampled were higher in spring 2010 (4500x) than in fall 2009 (2500x). While androgen equivalents were significantly higher at the outfall in spring 2010, all urban sites were similar in androgenic activity in fall 2009. Overall, all urban sites had significantly higher androgenic activity than the ranch site on both sampling dates. No anti-estrogenic or anti-androgenic activity was detected in any sample (data not shown).

### Water Chemistry

**Figure 11:**
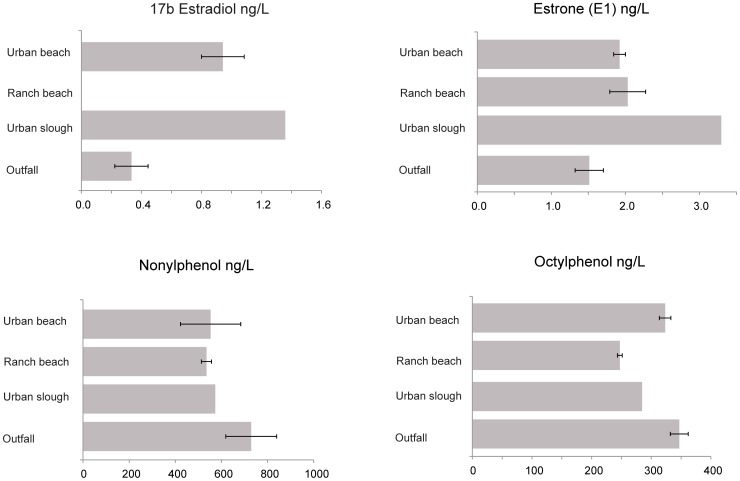
Hormone and alkylphenol water chemistry. Levels of 17 alpha and beta estradiol, estrone, testosterone, androstenedione, progesterone, estriol and the alkylphenols nonylphenol and octylphenol at ng/L were measured in grab samples from the wastewater outfall (Boynton Slough, *n* = 3), ranch beach (Denverton Slough, *n* = 2), urban slough (Peytonia Slough, *n* = 1), and urban beach (Suisun Slough, *n* = 2). Error bars represent standard error. Testosterone, androstenedione, progesterone, estriol, and 17α-estradiol concentrations were measured but were below detection or quantitation limits. Nonylphenol and octylphenol concentrations are estimated, levels measured were outside of the quantitative range. Bars indicate mean; error bars indicate 95% confidence intervals.

Water sampled from the urban and ranch beaches, urban slough (Peytonia) and the outfall (Boynton) contained detectable levels of 17-beta estradiol (E2), estrone (E1), nonylphenol (NP) and octylphenol (OP) (Figure 11). Levels of E2 and E1 were higher in the urban slough than at the outfall. Nonylphenol levels were highest at the outfall, but octylphenol was found at roughly equivalent concentrations at all sites sampled. Because only one sample was available for analysis from the urban slough, standard error could not be calculated for measurements from this site.

Nine moderately hydrophilic pesticides were detected in the grab water samples collected at PED deployment and retrieval (Table 2). Water samples from the municipal wastewater outfall/urban site contained: 3,4-dichloroaniline (3,4-DCA), atrazine, carbaryl, diazinon, fipronil, hexazinone, metolachlor, simazine, and trifluralin. At the ranch site all of these contaminants were also detected, with the exception of 3,4-DCA, diazinon, hexazinone, metolachlor, and trifluralin. Although a slightly higher concentration of atrazine was detected at one ranch sampling location in comparison to the municipal wastewater outfall, all other contaminants were at higher concentrations in water sampled from the vicinity of the outfall (Table 2).

**Table 2:** Pesticide, herbicide, and fungicide passive sampler and water chemistry.

Site	Dates	3,4-DCA	Atrazine	Bifenthrin	Carbaryl	Diazinon	Dieldrin
Ranch South	6/11/09–7/01/09	nd/nd	nd/2.0^a^	15.7±1.9/nd	nd/11.0±3.2	nd/nd	43.0±3.7/x
Ranch North	6/16/09–7/1/09	nd/nd	nd/3.4^a^	6.0±0.76/nd	nd/nd	nd/nd	49.4±7.4/x
Wastewater Outfall	8/04/09–8/17/09	nd/7.95±0.55	nd/2.9^a^	32.2±4.7/nd	nd/35.0±14.9	nd/9.3±4.8	30.9±3.9/x
		Fipronil	Hexazinone	Metolachlor	*p p*’ -DDD	*p p*’ -DDE	*p p*’ -DDT
Ranch South	6/11/09–7/01/09	5.9±1.04/nd	x/nd	nd/nd	3.2±0.50/nd	8.9±0.59/nd	3.7±0.65/nd
Ranch North	6/16/09–7/1/09	nd/nd	x/nd	nd/nd	3.3±0.65/nd	12.0±0.80/nd	4.8±0.59/nd
Wastewater Outfall	8/04/09–8/17/09	7.9±3.7/9.0±1.4	x/12.1^a^	nd/6.3±3.9	6.4±0.35/nd	24.3±2.2/nd	11.0±0.68/nd
		PCA	PCNB	Simazine	Trifluralin		
Ranch South	6/11/09–7/01/09	nd/nd	nd/nd	nd/16.4±4.0	4.0±1.2/nd		
Ranch North	6/16/09–7/1/09	nd/nd	nd/nd	nd/14.4±4.2	2.5±0.42/nd		
Wastewater Outfall	8/04/09–8/17/09	23.2±2.7/nd	7.1±0.46/nd	nd/43.0±1.5	5.7±0.23/2.1±0.70		

The first position in each cell is the amount detected in the PED in ng/PED (*n* = 4), and the second position in each cell is the amount detected in grab samples taken at the beginning and end of each PED deployment period in ng/l (*n* = 2). For concentrations with subscript a, a standard deviation could not be calculated since the chemical was only detected in one replicate.

An “nd” indicates that chemicals was analyzed for but not detected, “x” indicates that chemical was not analyzed for in that matrix. The following chemicals were analyzed for but were not detected: 3,5 DCA, butylate, clomazome, cycloate, cyfluthrin, cypermethrin, DCPA, deltamethrin, diazinon, EPTC, esfenvalerate, ethalfluralin, etofenprox, malathion, methidathion, methophrene, methylparathion, metolachlor, mokinate, napropramide, oxyfluorfen, pebulate, pendimethalin, permethrin, phenothrin, phosmet PBO, prometryn, propanil, propyzamide, remethrin, tau-fluvalinate, tefluthrin, tetramethrin, and thiobencarb.

Nine moderately hydrophibic pesticides (octanol-water partition coefficient, log K_ow_ >4) were detected in the PEDs deployed at the two sites in 2009. At the municipal wastewater outfall the following contaminants were detected from 4 August –17 August 2009: bifenthrin, dieldrin, fipronil, permethrin, *p,p*’ – DDD, *p,p’* – DDE, *p,p’* – DDT, PCA (pentachloroanisole), PCNB (pentachloronitrobenzene), and trifluralin (Table 2). These contaminants were also detected in PEDs deployed at the ranch site from 11 June or 16 June –1 July 2009, with the exception of PCA, PCNB, and permethrin. With the exception of the persistent legacy contaminant dieldrin, all contaminants detected in PEDs were found at higher amounts at the urban sites than at the ranch site. Hydrophilic chemicals that tend not to adsorb to substrates such as plastic or sediments (i.e. simazine) were found in water grab samples.

## Discussion

**Figure 12:**
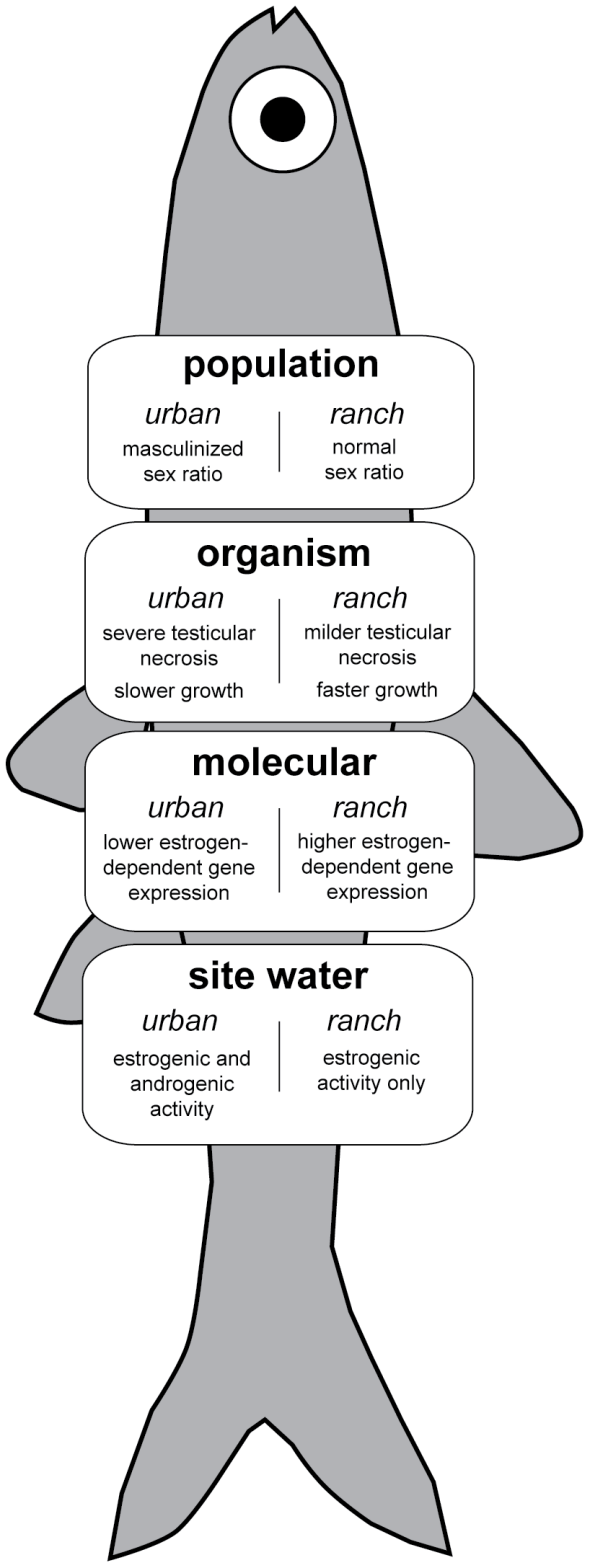
Conceptual model of multi-tiered approach and summary of results at increasing levels of biological scale in *Menidia audens*.

At this study’s initiation, we hypothesized that endocrine disrupting chemicals and effects would primarily be observed at the urban beach site, and that despite the presence of cattle at the ranch site, less evidence of endocrine disruption would be observed there. A true reference site was not sampled due to the lack of adequately unimpacted seining beaches with similar salinity and tidal regimes in the region. While EDCs were detected at both sites via the CALUX assay and chemical analyses, responses in fish at the ranch site were limited to changes at the molecular level (higher relative *Vtg* and *Chg* mRNA levels in males) while putative impacts at numerous levels of biological organization were observed in samples from the urban site (Figure 12).

### Site Water: Endocrine Activity and Chemistry

Although many studies have utilized the CALUX assay in the laboratory and in the field to measure endocrine activity in samples [49], [56], [57], few have attempted to link responses or contaminants in fish with the measured levels of endocrine activity detected in complex environmental samples [58]. The value of this effect-based approach is that it allows assessment of complex environmental mixtures and incorporates the activity of unknown EDCs unlikely to be identified by even extensive chemical analyses. Although the ER and AR CALUX used in this study were of human origin, it is known that both the ER and AR are highly conserved from lower to higher vertebrates [59]. Therefore, responses measured by the CALUX assay are relevant and would be expected to be comparable to *in vivo* responses in fishes.

Our findings of similar levels of estrogenic activity at both sites run counter to a number of studies that have reported high levels of estrogens or estrogenic effects in sites receiving municipal wastewater effluent compared to other sites [3], [60]. We detected very low levels of natural estrogens at both the urban/municipal wastewater outfall site and the ranch site, and although levels of estrogenic contaminants such as DDT, DDE, DDD, and bifenthrin were also detected and were present at higher levels at the urban site than the ranch site, overall estrogenic activity did not largely differ. Estradiol equivalents were lower in the spring sample, likely due to dilution from winter rains.

In contrast, testosterone equivalents were lower in the fall in comparison to the spring, which could indicate that androgenic contaminants originate from different sources than estrogenic contaminants. Other studies have found that androgens and androgenic compounds comprise a sizeable proportion of the EDCs present in treated municipal wastewater effluent [61], [62], which supports our findings. Notably, a diverse array of compounds, from pesticides to PAHs, can act as androgen agonists depending on the concentration [63]. For example, both atrazine and simazine, which were detected in municipal wastewater samples (simazine at much higher levels at the urban site), have demonstrated *in vitro* androgenic activity [64]. The AR CALUX assay may have also been sensitive to natural and synthetic glucocorticoids and progestins present as pharmaceuticals in treated municipal wastewater effluent, since the assay may not discriminate well between these three types of compounds because the DNA binding site for each of these steroid receptors is identical [56]. Both glucocorticoids and progestins are now known to masculinize fishes [1]. Considering that the natural androgens testosterone and androstenedione were not detected, the contribution of xenoandrogens and other EDCs to AR CALUX activity at the urban site may be significant.

### Gene Expression

Males caught from the ranch site that had been exposed to water with measured estrogenic activity expressed transcripts for both *Vtg* and *ChgL*, as has been found in other species of fish exposed to estrogenic environmental mixtures [1], [3], [12]. However, males from the urban site had significantly lower levels of *Vtg* and *ChgL* mRNA, even though both sites had roughly equivalent levels of estrogenic activity. Levels of mRNA for the same estrogen-responsive genes in females did not differ significantly between sites, although those data followed the same trend as in males (higher mRNA levels of estrogen-responsive genes at the ranch site), suggesting that the lack of significance was due to low statistical power rather than an opposite pattern of expression in females.

The observed difference in expression of these genes in males and females between the ranch and urban sites could be attributed to the presence of androgenic EDCs (detected by the AR CALUX) at the urban site. For example, female fish (*Pimephales promelas, Danio rerio, Oryzias latipes*) exposed to androgenic EDCs exhibited reduced Vtg protein levels expression relative to controls [65]. Studies targeting fish gonads have found that additions of androgens to ovarian tissue cause a dose-dependent decrease in estradiol production [66], which would lead to a decrease in or absence of Vtg production. Lowered expression of estrogen-responsive genes in urban females during the reproductive season could indicate masculinization due to androgenic EDC exposure, and potentially lowered fecundity, but differences between females from the two sites were not significant. In future studies larger sample sizes should be collected to account for the large amount of variability inherent in wild populations of fish, as mRNA levels varies widely with age, reproductive status and environmental conditions. It should also be noted that it is difficult to make a direct comparison between ER and AR activity in grab samples, which record a snapshot of EDC presence on a single day, and transcript levels in fish exposed over a prolonged period, during which EDC concentrations in the water column and other environmental conditions could vary widely.

### Gonad Health

Gonad health in *M. audens* was evaluated using two endpoints, gonadal somatic index (GSI) and histology. The appearance of histological sections, coupled with analysis of *Vtg* expression, is considered one of the most sensitive endpoints for determining whether endocrine disruption has occurred [67]. Exposure to both estrogenic and androgenic compounds has been shown to reduce GSI and increase the incidence of germ cell necrosis, which may represent an interruption of spermatogenesis [40], [68], [69]. Notably, moderate levels of necrosis were higher (marginally significant) and severe cases of necrosis were significantly higher and GSI in males was significantly lower at the urban beach, at which we detected the presence of both estrogenic and androgenic compounds. Both lowered GSI and increased necrosis could result in lowered sperm count and hence lowered fecundity of urban males.

### Growth

Although measurement of growth rate using otoliths is rarely performed in toxicological studies, they have been shown in one study to be a more sensitive measure of growth than merely focusing on somatic changes [70]. To our knowledge, otoliths have not been used to compare the growth rates of fish exposed to different types of EDCs, which may modify growth via interactions with the insulin-like growth factor system [71].

A recent study found that the length, body mass and growth rate of sticklebacks (*Gasterosteus aculeatus*) influenced by municipal wastewater effluent were greater than that of reference populations [72]. Our results were more mixed. While we found that males caught from the urban site were significantly longer than males caught from the ranch site, examination of otoliths revealed that the growth rate of urban males was significantly slower than ranch males. Urban males were also significantly larger than urban females, which is surprising considering that in *Menidia* populations female fecundity is strongly correlated with increasing SL and weight [33]. This is another indication, along with significantly reduced male GSI and a significantly higher incidence of severe testicular necrosis, that overall fecundity may be lower at the urban/municipal wastewater outfall site.

The difference in overall growth rate could be due to differences in food availability between the two sites. However, this cannot explain males being significantly larger than females (in standard length) at the urban site. It is possible that because *M. audens* likely have temperature sensitive sex determination to some extent [33], that some fish born in the early spring that were genotypically female (more females produced at colder temperatures) actually became phenotypically male due to early life exposure to androgenic EDCs. As a result, these early season urban males had a longer period to grow than ranch males born later in the year, so regardless of the slower growth rate urban males were significantly longer overall.

### Population Level

While the expected pattern of higher numbers of female fish was observed at both the urban and ranch sites, the urban site had a significantly lower proportion of females throughout the entire breeding season in both 2009 and 2010. Additionally, although more females are produced earlier in the year, the population should even out to approximately 50% female, 50% male when census data are aggregated across the entire year [73]. Hence, it appears that the population exposed to urban run-off and treated municipal wastewater effluent, which has been demonstrated via the CALUX assay to contain significantly higher amounts of androgens or xenoandrogens, may be undergoing masculinization.

Results of field exposures are also not always clear-cut due to the presence of complex environmental mixtures. For example, studies conducted downstream of municipal wastewater or pharmaceutical discharges have shown simultaneous expression of *Vtg* in male fish (i.e., feminization) and male biased sex ratios (i.e. masculinization) in the same population [74], [75]. Even compounds that are considered to be estrogenic, such as nonylphenol, can exert unexpected effects at the population level. For example, Japanese medaka (*Oryzias latipes*; also in the superorder Atherinomorpha with *M. audens*), exposed to high concentrations of nonylphenol had a decreased proportion of females in comparison to controls [76]. Furthermore, compounds found in municipal wastewater effluent, such as glucocorticoids and the synthetic progestins levonorgestrol and norethindrone, have been shown to masculinize and reduce the fecundity of various fish species [77], [78], [79]. Masculinization can also occur following exposure to hypoxic events in the Atlantic croaker, *Micropogonias undulatus* [80], and hypoxia is known to occur in Suisun Marsh [81], particularly in the vicinity of the urban beach. The masculinization of *Menidia audens* may have occurred due to the interplay of several of these dynamics.

### Summary

The need for EDC studies that examine and link impacts at multiple biological scales, including the population level, have been suggested by several recent reviews in the field [3], [82], [83] and such studies are becoming increasingly common. For example, lab studies with the fathead minnow and other species have been used to predict population trajectories [10], [11], [84] and changes in reproductive biomarkers have been linked to land use (urbanization) and to exposure to complex environmental mixtures in the lab [12], [14]. To date, the most direct link between lower level molecular endpoints (*Vtg* expression) and population persistence has been demonstrated in a study undertaken by Kidd et al. [9], who observed a population crash in fathead minnows exposed for multiple years to part per billion levels of ethinylestradiol in an experimental lake. Our study expands upon these efforts that primarily utilized exposures to known chemicals or lab exposures with standard test species by evaluating the impact of environmental mixtures with measured levels of endocrine activity in the field on several tiers of the biological hierarchy in wild fish. It encompasses endpoints with both high ecological significance (sex ratio, growth) and high mechanistic significance (gene expression, histopathology) [83].

In our study, links were observed between the biological scales examined for signs of endocrine disruption in *M. audens*. At the ranch site, where primarily estrogenic compounds were present, males had significantly higher expression of estrogen-responsive genes. At the urban/municipal wastewater outfall site, which is contaminated by both estrogens and comparatively higher concentrations of androgens, both males and females had relatively low expression of estrogen-responsive genes, males had significantly lower GSI and a significantly higher incidence of severe testicular necrosis, and the proportion of females caught throughout the spawning season was low compared to the ranch site and in comparison to observations of *Menidia* sex ratios in other populations.

Additionally, it was found that males at the urban site were significantly larger (in SL) than females. This finding runs counter to the reproductive strategy of most atherinid fishes, in which females are larger than males, maximizing their capacity to carry oocytes [28], [33], [73]. Males were larger despite having a slower growth rate than urban females, which may be due to genetically female larval *M. audens* being masculinized early in the season, when colder temperatures should result in a female-biased population. Future research will seek to confirm whether this is occurs experimentally, both in the laboratory and in the field, using genetic markers of sex determination. The sequencing of the genome of several *Menidia* species and subsequent development of a microarray, now underway, will allow the mechanisms that underlie endocrine disruption to be addressed (R.E. Connon and S.M. Brander, unpublished data). Additionally, the current development of laboratory approaches that measure fertilization success (spawning trials) and a population dynamic model, both of which quantify the impact of altered sex ratio on reproductive output, will better inform efforts to deduce the potential for impacts at the population level (S.M. Brander, R.E. Connon and J.W. White, unpublished data). It may be possible to extrapolate results from ongoing work to other estuarine species that are either endangered or more difficult to sample. Future lines of research should continue to follow populations of *M. audens* at sites in the San Francisco Bay region to monitor long-term trends in sex ratio, gonad health, and gene expression in relation to EDC activity. However, considering the ubiquity of *Menidia* species, this multi-tiered approach could potentially be expanded to estuaries nationwide.

## Supporting Information

Table S1 **Results of logistic regression (binomial error) on sex ratios.**
(DOCX)

Table S2 **Results of linear regression on male gonadosomatic index.**
(DOCX)

Table S3 **Results of linear regression on female gonadosomatic index.**
(DOCX)

Table S4 **Summary of fixed effects from mixed-model linear regression on otolith increment width.**
(DOCX)

Table S5 **Results of linear regression on male standard length.**
(DOCX)

Table S6 **Results of linear regression on female standard length.**
(DOCX)

Table S7 **Results of linear regression on standard length (SL) of both sexes.**
(DOCX)

Appendix S1 **Supplemental methods for CALUX assay and chemical analysis.**
(DOCX)
