# Supplementary material for: From ‘Omics to Otoliths: Responses of an Estuarine Fish to Endocrine Disrupting Compounds across Biological Scales
Source: PLoS One. 2013 Sep 25;8(9):e74251. doi: 10.1371/journal.pone.0074251 (PMC3783432; doi:10.1371/journal.pone.0074251)
Supplement: Table S1 — Results of logistic regression (binomial error) on sex ratios. (DOCX) [file pone.0074251.s001.docx]

Table S1. Results of logistic regression (binomial error) on sex ratios (proportion female; *n* = 282)

| **Effect** | **Estimate** | **SE** | ***p*** |
| --- | --- | --- | --- |
| Intercept | 0.583 | 0.472 | 0.2167 |
| Site (urban) | -1.098 | 0.305 | 0.0003 |
| Year (2010) | -0.624 | 0.339 | 0.0654 |
| Julian date | -0.007 | 0.003 | 0.0170 |

Notes: Site and Year were treated as categorical effects; the ranch site in 2009 was considered the baseline treatment. All interaction effects had *p* > 0.1 and were discarded from model. SE = standard error.
