# Supplementary material for: From ‘Omics to Otoliths: Responses of an Estuarine Fish to Endocrine Disrupting Compounds across Biological Scales
Source: PLoS One. 2013 Sep 25;8(9):e74251. doi: 10.1371/journal.pone.0074251 (PMC3783432; doi:10.1371/journal.pone.0074251)
Supplement: Table S2 — Results of linear regression on male gonadosomatic index. (DOCX) [file pone.0074251.s002.docx]

Table S2. Results of linear regression on male gonadosomatic index (GSI) (*n* = 135)

| **Effect** | **Estimate** | **SE** | ***p*** |
| --- | --- | --- | --- |
| Intercept | 8.501×10^-2^ | 8.967×10^-3^ | < 2×10^-16^ |
| Site (urban) | -2.014×10^-2^ | 8.150×10^-3^ | 0.0148 |
| Year (2010) | -4.356×10^-2^ | 8.468×10^-3^ | 9.84×10^-7^ |
| Julian date | -2.676×10^-4^ | 4.650×10^-5^ | 6.08×10^-8^ |
| Site (urban) × Julian date | 7.080×10^-5^ | 4.145×10^-5^ | 0.09 |
| Site (urban) × Year (2010) | 6.392×10^-3^ | 3.912×10^-3^ | 0.10 |
| Year (2010) × Julian date | 2.124×10^-4^ | 4.375×10^-5^ | 3.46×10^-6^ |

Notes: Site and Year were treated as categorical effects; the ranch site in 2009 was considered the baseline treatment. All interaction effects with *p* > 0.1 were discarded from model. SE = standard error.
