# Supplementary material for: From ‘Omics to Otoliths: Responses of an Estuarine Fish to Endocrine Disrupting Compounds across Biological Scales
Source: PLoS One. 2013 Sep 25;8(9):e74251. doi: 10.1371/journal.pone.0074251 (PMC3783432; doi:10.1371/journal.pone.0074251)
Supplement: Table S3 — Results of linear regression on female gonadosomatic index. (DOCX) [file pone.0074251.s003.docx]

Table S3. Results of linear regression on female gonadosomatic index (GSI) (*n* = 35)

| **Effect** | **Estimate** | **SE** | ***p*** |
| --- | --- | --- | --- |
| Intercept | 7.228×10^-2^ | 3.497×10^-2^ | 0.047 |
| Site (urban) | 1.231×10^-2^ | 1.110×10^-2^ | 0.276 |
| Year (2010) | 1.250×10^-2^ | 1.258×10^-2^ | 0.328 |
| Julian date | 1.492×10^-4^ | 1.988×10^-4^ | 0.458 |

Notes: Site and Year were treated as categorical effects; the ranch site in 2009 was considered the baseline treatment. All interaction effects with *p* > 0.1 were discarded from model. SE = standard error.
