# Supplementary material for: From ‘Omics to Otoliths: Responses of an Estuarine Fish to Endocrine Disrupting Compounds across Biological Scales
Source: PLoS One. 2013 Sep 25;8(9):e74251. doi: 10.1371/journal.pone.0074251 (PMC3783432; doi:10.1371/journal.pone.0074251)
Supplement: Table S4 — Summary of fixed effects from mixed-model linear regression on otolith increment width. (DOCX) [file pone.0074251.s004.docx]

Table S4. Summary of fixed effects from mixed-model linear regression on otolith increment width.

| **Effect** | **Estimate** | **SE** | ***p*** |
| --- | --- | --- | --- |
| Intercept | 27.717 | 29.889 | 0.353 |
| Age | 22.781 | 0.190 | < 0.0001 |
| Sex (male) | -10.788 | 15.422 | 0.484 |
| Site (urban) | -49.812 | 19.279 | 0.010 |
| Age × Sex (male) | -0.617 | 0.263 | 0.019 |
| Age × Site (urban) | 0.874 | 0.324 | 0.007 |
| Sex (male) × Site (urban) | 52.829 | 23.041 | 0.022 |
| Age × Sex (male) × Site (urban) | -3.384 | 0.390 | < 0.0001 |

Notes: Sex and Site were treated as categorical effects; ranch females were considered the baseline treatment. SE = standard error.
