# Supplementary material for: From ‘Omics to Otoliths: Responses of an Estuarine Fish to Endocrine Disrupting Compounds across Biological Scales
Source: PLoS One. 2013 Sep 25;8(9):e74251. doi: 10.1371/journal.pone.0074251 (PMC3783432; doi:10.1371/journal.pone.0074251)
Supplement: Table S5 — Results of linear regression on male standard length. (DOCX) [file pone.0074251.s005.docx]

Table S5. Results of linear regression on male standard length (SL) (*n* = 205)

| **Effect** | **Estimate** | **SE** | ***p*** |
| --- | --- | --- | --- |
| Intercept | 64.622 | 1.986 | < 2×10^-16^ |
| Site (urban) | 5.908 | 1.162 | 8.36×10^-7^ |
| Year (2010) | -3.193 | 1.236 | 0.010 |
| Julian date | -0.040 | 0.011 | 4.47×10^-4^ |

Notes: Site and Year were treated as categorical effects; the ranch site in 2009 was considered the baseline treatment. All interaction effects with *p* > 0.1 were discarded from model. SE = standard error.
