# Supplementary material for: From ‘Omics to Otoliths: Responses of an Estuarine Fish to Endocrine Disrupting Compounds across Biological Scales
Source: PLoS One. 2013 Sep 25;8(9):e74251. doi: 10.1371/journal.pone.0074251 (PMC3783432; doi:10.1371/journal.pone.0074251)
Supplement: Table S6 — Results of linear regression on female standard length. (DOCX) [file pone.0074251.s006.docx]

Table S6. Results of linear regression on female standard length (SL) (*n* = 77)

| **Effect** | **Estimate** | **SE** | ***p*** |
| --- | --- | --- | --- |
| Intercept | 70.342 | 3.914 | < 2×10^-16^ |
| Site (urban) | -0.646 | 2.875 | 0.823 |
| Year (2010) | -6.528 | 3.409 | 0.059 |
| Julian date | -0.046 | 0.023 | 0.055 |

Notes: Site and Year were treated as categorical effects; the ranch site in 2009 was considered the baseline treatment. All interaction effects with *p* > 0.1 were discarded from model. SE = standard error.
