# Supplementary material for: From ‘Omics to Otoliths: Responses of an Estuarine Fish to Endocrine Disrupting Compounds across Biological Scales
Source: PLoS One. 2013 Sep 25;8(9):e74251. doi: 10.1371/journal.pone.0074251 (PMC3783432; doi:10.1371/journal.pone.0074251)
Supplement: Table S7 — Results of linear regression on standard length (SL) of both sexes. (DOCX) [file pone.0074251.s007.docx]

Table S7. Results of linear regression on standard length (SL) of both sexes (*n* = 282)

| **Effect** | **Estimate** | **SE** | ***p*** |
| --- | --- | --- | --- |
| Intercept | 65.076 | 1.946 | < 2×10^-16^ |
| Sex (female) | 4.076 | 1.627 | 0.013 |
| Site (urban) | 5.853 | 1.310 | 1.15×10^-5^ |
| Year (2010) | -3.886 | 1.237 | 0.002 |
| Julian date | -0.041 | 0.010 | 9.81×10^-5^ |
| Sex (female) × Site (urban) | -6.535 | 2.622 | 0.013 |

Notes: Site and Year were treated as categorical effects; ranch male was considered the baseline treatment. All interaction effects with *p* > 0.1 were discarded from model. SE = standard error.
