# Supplementary material for: From ‘Omics to Otoliths: Responses of an Estuarine Fish to Endocrine Disrupting Compounds across Biological Scales
Source: PLoS One. 2013 Sep 25;8(9):e74251. doi: 10.1371/journal.pone.0074251 (PMC3783432; doi:10.1371/journal.pone.0074251)
Supplement: Appendix S1 — Supplemental methods for CALUX assay and chemical analysis. (DOCX) [file pone.0074251.s008.docx]

Appendix S1. Supplemental methods for CALUX assay and chemical analysis.

*CALUX*

As described in Giudice and Young 2010, all glassware was washed with soap and acetone and baked in a muffle furnace at 232º C prior to use. Prior to extraction samples were shaken thoroughly to evenly distribute any suspended solids and poured into a filter holder set-up 250 ml at a time (Fisher Scientific, Waltham, MA, USA), creating two replicates for each grab sample. Samples were extracted under 15-20 mm Hg vacuum pressure through a 934-AH Whatman filter. Following extraction, samples were acidified to pH 2 with 10N HCl to improve the binding of EDCs with hydroxyl functional groups to the C-18 cartridge. A 24-cartridge Supelco solid phase extraction manifold (Supelco, Bellefonte, PA, USA) attached to a vacuum flask was used to extract filtered samples through OASIS HLB 6 cc cartridges (Waters, Milford, MA, USA). Cartridges were pre-conditioned using 5 ml each of EtAc:AOc (75:25), 5 mLs MeOH and 5 ml acidified MilliQ water. Samples were extracted over an hour-long period at 5 mm Hg. Prior to elution, cartridges were washed twice with 5 ml 5% MeOH. Cartridges were then eluted twice with 4 ml EtAc:AOc (75:25) into glass tubes and dried under a nitrogen evaporator at 60-70º C.

*Chemical Analysis: Quality Assurance*

Pesticide concentrations in water and PEDs were validated against a comprehensive set of performance based quality assurance/quality control (QA/QC) criteria including laboratory blanks, matrix spikes, and surrogate recovery. One laboratory water blank was processed to test the cleanliness of the laboratory procedures. No pesticides were detected in any of the blank samples. Ring-^13^C_3_-atrazine and diethyl-d_10_ diazinon were used as recovery surrogates to assess the efficiency of sample extraction. Percent recovery of surrogates for all samples analyzed (including QC samples) ranged from 77% to 101% with a mean (+ standard deviation) of ring-^13^C_3_-atrazine and diethyl-d_10_ diazinon of 90 + 6 % and 90 + 7 %, respectively. Two water samples were spiked in the laboratory with pesticides and the percent recovery ranged from 82 to 110% with a median of 92%.

No pesticides were detected in the two blanks processed with the PED samples. Percent recoveries of the surrogates, ring-^13^C_12_-*p,p’* DDE and phenoxy-^13^C_6_-*cis*-permethrin ranged from 72 to 101 %. The mean (+ standard deviation) of ring-^13^C_12_-*p,p’* DDE and phenoxy-^13^C_6_-*cis*-permethrin of 88 + 8% and 87 + 9 %, respectively. Two samples were spiked with pesticides and processes with PED samples and the percent recovery of matrix spiked samples ranged from 70 to 99 % with a median of 85 %.

*Chemical Analysis: Determination of Detection Limits*

Surface-water method detection limits (MDLs) were validated for the majority of the pesticides previously (Hladik et al., 2008) using the EPA procedure described in 40 CFR Part 136 (U.S. Environmental Protection Agency, 1992). Water samples used to determine MDLs were collected in 2005 from the Sacramento River at Miller Park. MDLs for all compounds in water ranged from 0.9 to 10.1 ng/L and instrumental limits of detection (LOD) ranged from 0.5 to 1.0 ng/L (Table B.1, Appendix B). Analytes detected at concentrations greater than the instrumental LOD but less than the MDL were reported as estimates. Instrumental LODs for pesticides measured in PEDs ranged from 5 to 10 ng/PED (Table B.2, Appendix B).

*Chemical analysis: Instrumental analysis*

All sample extracts (1 µL injection volume) were analyzed on an Agilent 5975 gas chromatograph (GC)/electron ionization mass spectrometer (EI-MS) (Folsom, CA, USA). Analyte separation on the GC was achieved using a 30 m x 0.25 mm i.d., 0.25 μm DB-5ms fused silica column (Agilent Technologies, Folsom, California) with helium as the carrier gas. The temperature of the splitless injector was held constant at 275º C. The temperature program was 80º C (hold 0.5 min), increase to 120º C at 10º C/min, increase to 200º C at 3º C/min (hold 5 min), followed by a third increase to 219º C at 3º C/min, and a final increase to 300º C at 10º C/min (hold 10 min). The transfer line, quadrupole and source temperatures were 280º C, 150º C and 230º C, respectively. Data for all pesticides was collected in selective ion monitoring mode (SIM) with each compound having one quantifier and 1-2 qualifier ions (Table B.1, Appendix B). Instrument calibration was achieved using calibration concentration standards that spanned the linear range of instrument response. Calibration curves were considered acceptable if the *r*^2^ for each individual compound was greater than 0.995. The responses of the instrument was monitored every 6-8 samples with mid-level check standards. The instruments were considered to be stable if the recovery of the check standards fell within the range of 80-115 % of the nominal standard concentration. If environmental sample concentrations fell outside the linear range of the instrument, the samples were diluted appropriately and re-analyzed.
